# Personal perspectives in the life sciences for the Royal Society's 350th anniversary

**DOI:** 10.1098/rstb.2009.0232

**Published:** 2010-01-12

**Authors:** Georgina Mace

2010 is the 350th anniversary of the Royal Society. The *Philosophical Transactions of the Royal Society*, first published in 1665, while being a few years younger than the society itself, is still the oldest scientific journal printed in the English-speaking world and the world's longest running scientific journal in continuous production. Our authors have included many of the most outstanding scientists of the times including Isaac Newton, Michael Faraday and Charles Darwin, and the contents have communicated many of the major scientific findings of the past few centuries. Since 1887, the journal has been published as two separate publications, one serving the physical sciences and this one focusing on the life sciences.

The question of what we should do for the 350th anniversary of the society has preoccupied the editorial team since early 2008. One idea, quite common for scientific publishing anniversaries, was that we should identify key papers that had been published in the journal during its long history and republish them with commentary from a contemporary specialist, bringing the science up to date. Another idea was to focus specifically around a small number of contemporary controversies. However, we agreed that while the 350th anniversary is an important historical moment for the Royal Society, the moment was right to consider the state of the science and its future directions rather than simply to celebrate important historical findings. Our intention then was to produce an issue that would be forward-looking, providing a resource for the present and future more than a record of the past. We decided that a good way to do this would be to invite key thinkers on the contemporary topics of great interest and importance to review where their field was situated, give their perspectives and try to point to some promising as well as less promising routes for the future.

What are the contemporary topics of high interest and importance? There are various possible means to identify these, but our deliberations were given a helpful boost by a survey the Royal Society undertook in late 2007, asking fellows and university research fellows to briefly indicate what they thought were the ‘biggest gaps in knowledge’. The group polled is probably neither random nor well sampled but can at least be regarded as well informed and relevant for the task. Their responses were unsurprisingly divergent and also had a tendency to be either very specialized or very general. Rather alarmingly, or perhaps rather charmingly, many wrote about the current issues in their own particular research area. But there was also quite a strong convergence of views from across the wide range of specialisms sampled, towards just four or five major topics in the life sciences. Probably, the most common were the topics to do with complex biological systems, especially the brain and genetic control of organism function. Many respondents cited questions related to brain function; how the mind relates to the brain; human and animal cognition, consciousness and the emerging links to neurobiology. A related set of topics concerning the nature of intelligence, biological information processing and the way in which artificial intelligence systems can help us to understand complex biological processes were also common. A second set of topics raised by many concerned genome to organism processes, ways in which the emerging technologies associated with sequencing and bioinformatics might contribute to our understanding of the way that the genome controls the functioning of organisms. Also commonly raised was the long standing but still unresolved set of questions about the origins of life and the sources and maintenance of variability. Finally, and notably, the more commonly mentioned by the junior research fellows was a set of topics around environmental change, human population growth, sustainability and the future of life on Earth.

Using this set of topics as a starting point, we identified leading researchers working across the biological sciences, but especially in these areas. We invited them to consider the big questions in the broad field in which they work, to identify new or promising approaches as well as the aspects of research where they were sceptical about the current and conventional wisdom. Another non-random sorting then took place as different people accepted or declined the offer, but the final collection is pretty well balanced across the key topics.

The order in which the papers are presented starts with the set of issues and problems related to sustainable development in the face of environmental degradation, failing policies and changing human demography. A closely inter-related set of papers point to the intricate linkages between human societal norms and structures, and the continuing spiral of environmental degradation. Working our way out of this will require integrated solutions across the social, economic and environmental sciences. A poignant fact noted by several authors here is that just at the moment when we most need cooperative human behaviour, cultural and economic processes associated with economic growth and development are leading to the breakdown of the community sizes and structures most likely to deliver what is needed. Dasgupta introduces the idea of natural capital as a necessary consideration in addition to conventional economic measures used to denote the well-being of societies, and recommends that this be routinely used to assess sustainability. Levin also focuses on sustainability but from the perspective of the biological forces that determine cooperation as opposed to competition. His conclusion on the overriding importance of cooperation is picked up in more detail by Nowak in his general analysis of the evolution of cooperation, especially in the case of spatially or demographically structured populations. These papers, each based on the fundamental principles from distinct scientific areas, individually and collectively point to the significant areas that can inform contemporary debates about the sustainability of human societies.

The urgency of the problem is highlighted by Mooney who documents the degradation of biological systems and of ecosystem services upon which we depend. While the evidence of driving processes and possible solutions is becoming clearer, there are blockages to progress that seem to sit at the interface of the science and policy worlds. May describes the ecological issues relating to biodiversity loss in the face of continuing pressures from land for food production, energy, population trends and climate change. How can all these different demands be accommodated, and how much is the society willing to accept technological solutions? Even if those solutions do exist and function successfully in the narrow context in which they are developed, what might be their unintended side effects or wider consequences?

Scientific progress will undoubtedly contribute new solutions. These may come from new science and technology or from new applications from established disciplines. Loreau argues for a more coherent ecosystem ecology that brings the intricate processes that ecology has revealed to bear on resolving the ecosystem service failures that result from environmental degradation. Beddington assesses impending agricultural and land-use demands with a look at new technologies, and Hill examines what quantitative genetics, which essentially gave us the tools for the first agricultural revolution based on selective breeding, can deliver in the new genomics era.

Hill's paper neatly provides a link between the environmental and the applied problems to the suite of fundamentally interesting issues to do with the origins of life and genetic diversity, the diversification of life and the predictability of evolution. In this area, there has been massive progress in recent times, based partly on the new discoveries but also on new technologies and experimental systems. One general conclusion that emerges from this set of papers, most appropriately in the current celebrations related to Darwin's anniversary, is the progress in understanding the shape of the history of life and the role of key innovations in permitting adaptive evolution. Bell addresses the tempo and mode of evolution, especially the conflicting views on whether slow and gradual evolution can really be the norm given the recent genetic- and field-based evidence for strong selection and rapid, major changes. Evolutionary novelty and its potential to influence the nature of diversification and the appearance of novelties is then detailed by Barrett, for plant reproductive traits, and by Cavalier-Smith for the major transformations in the history of life. The major transitions that underpin both these papers are discussed in more general terms by Conway-Morris who presents the evidence for randomness and open-endedness in evolution, and concludes that it is more predictable than generally supposed.

A human demographic shift of particular interest to many is the current and future shifts to an ageing population. Linda Partridges' paper provides the link between evolutionary biology and emerging techniques for medical intervention. An evolutionary look at ageing clearly points to the multi-disciplinary nature of the associated health problems. Of enormous topical relevance, Watt then explains why stem cell therapies may be of particular relevance across a range of medical problems affecting both old and young.

Frith then discusses the emerging links between neuroscience and social cognition; surely a key area for future research, where a common approach to understanding the brain is to examine function across a range of social processes and situations. An alternative approach described by Hinton is to develop computer systems based on biology to help us understand the most complex processes such as visual processing.

Finally, but by no means least, are two papers taking different approaches to the emerging genomics revolution where technologies are providing enormous amounts of new data to inform scientific understanding of genetic control. O'Brien introduces us to some of the many benefits from this new technology and some emerging patterns; Brenner reminds us that sometimes small reductionist experiments can provide clearer clues to process than mass processing.

There is much to contemplate in these papers, and many valuable insights. I thank all the authors for their willingness to think deeply and broadly, and communicate important and complex processes so clearly. A measure of the success of this volume would be to see the progress made in these the next time the society or the journal has a major anniversary. Thanks are also due to the Editorial team, especially Claire Rawlinson and James Joseph, as well as the members of the journal's Editorial Board for their suggestions and their ideas that shaped this issue.

